# Mitochondrial response to fever boosts T_H_1-driven inflammatory responses

**DOI:** 10.1097/IN9.0000000000000058

**Published:** 2025-03-13

**Authors:** Gillian Dunphy, David Sancho

**Affiliations:** 1 Immunobiology Laboratory, Centro Nacional de Investigaciones Cardiovasculares (CNIC), Madrid, Spain

**Keywords:** mitochondria, fever, inflammation, T_H_1 immunity

## Abstract

Increased body temperature, both locally and systemically, is a key feature of the inflammatory response. Heat is associated with increased blood flow to affected areas and increased immune infiltrate, yet increased temperature has also been described to have direct effects on immune cell function. In a recent study, Heintzman, et al investigated the effect of febrile temperature (39 °C) on T cell function. They describe increased T_H_1 function and fitness accompanied by a decrease in regulatory T cell suppressive function. These findings add another important consequence to our understanding of fever responses.

Upon infection, innate immune cells and barrier tissues respond to pathogen and damage-associated molecular patterns to induce inflammatory mediators, some of which are pyretic—they induce fever. During this acute fever response, DCs travel to the nearest afferent lymph node to present antigen to cognate T cells to initiate the adaptive immune response. CD4^+^ T cells, known as helper T cells (T_H_), are key both in the formation and the termination of adaptive immune responses. Presentation of antigen on MHCII along with costimulatory molecule binding stimulates TCR signaling on T_H_ cells, inducing a potent activation characterized by proliferation and cytokine production. Depending on the signals provided by the DC and in the microenvironment, T_H_ polarization into one of various subtypes is induced ^[1]^. The T_H_1 subtype differentiates in response to IL-12 and is defined by the production of IFNγ and responsiveness to intracellular bacteria. T_H_17 cells differentiate in response to IL-6 and TGFβ, produce IL-17, and are associated with antifungal responses. Induced regulatory T cells (iTregs) on the other hand, differentiate in response to TGFβ and are essential for inhibiting inflammatory responses and preventing inappropriate immune cell activation that can result in autoimmunity.

T_H_ cell activation requires extensive metabolic adaptations to support increased cellular function ^[2,3]^. These processes can be affected by the microenvironment, including nutrient stress and pH, as well as temperature ^[4]^. A T cell in a draining LN will compete for nutrients during its demanding clonal expansion, and the increase in glycolysis in these cells increases lactate production, decreasing pH levels, inhibiting continued T cell effector function ^[5]^. However, the effect of febrile conditions on immune cell function is only beginning to be explored. Heat, alongside pain, swelling, redness, and loss of function, is a cardinal feature of inflammation. This increase in body temperature, both local and systemic, can combat infection by decreasing bacterial and viral proliferation; however, physiological febrile temperatures (in the range of 38–39.5 °C) have also been reported to have direct effects on immune cell function ^[6]^. In the context of adaptive immunity, fever was shown to increase T lymphocyte trafficking via heat shock protein (HSP)-induced integrin expression ^[7]^. CD8^+^ T cell effector cytokine production has been shown to be positively regulated by increased physiological temperatures ^[8,9]^. And in the case of CD4^+^ T cells, studies have reported increases in T_H_17 cytokine production ^[10]^, and T_H_2 cytokine production ^[11]^. The polarizing conditions and incubation time at increased temperatures vary between these studies.

To separate the effect of increased temperature from the many other signals that are present upon acute infection, the authors of a recent study in *Science Immunology*, cultured CD4^+^ T cells in polarizing conditions for 2 days at 37 °C or 39 °C and compared functional readouts ^[12]^. They found that in T_H_1 cells but not in T_H_17 cells, there was decreased mitochondrial complex I (CI) activity when cultured at 39 °C. This led to disrupted forward electron transport and the generation of mitochondrial reactive oxygen species (mtROS), which induced oxidative DNA damage, upregulated the HSP70, and increased p53 activity selectively in T_H_1 cells, with these effects inversely correlating with the survival in these cells (Figure 1). Although survival was reduced in T_H_1 cells at 39 °C, the surviving cells displayed increased levels of IFNγ production, which was dependent on activation of the cyclic GMP-AMP synthase (cGAS)-stimulator of interferon genes (STING) pathway of cytosolic DNA sensing. Oxidized DNA has previously been shown to potentiate STING-dependent signaling and type I IFN (IFN-I) production in response to DNA damage ^[13]^. In line with this, ROS induction in T_H_1 cells at 39 °C led to a cGAS-STING-dependent induction in both IFN-I and IFNγ (Figure 1). However, this study identified a requirement for STING in the induction of ROS and DNA damage, indicating an additional upstream role for DNA sensing in the process of ROS induction at 39 °C.

A key finding of this article is that CI is temperature sensitive in T_H_1 but not in other Th subsets. Upon culture at 39 °C, the CI activity in T_H_1 cells decreases, but this is not observed in T_H_17 cells under the same conditions. The decrease in forward electron transport via CI was identified as the underlying cause for the increased ROS detected in cells cultured under febrile conditions. Oxidation of respiratory substrates in the mitochondria releases energy, which is used to generate ATP. However, a sizeable proportion of this energy is released in the form of heat. Indeed, the temperature of the mitochondria in fibroblast cell lines was found to be 50 °C even when the external temperature was 38 °C ^[14]^. At these temperatures, mitochondrial complexes were found to be stable ^[15]^. Given the stability of mitochondrial complexes at these temperatures, it is possible that the instability of CI in T_H_1 cells in febrile conditions may be indirect, due to an upstream factor that is sensitive to febrile temperatures. Interestingly, this study shows that T_H_1 cells, unlike T_H_17 and iTregs, do not show a relative increase in Glut1 expression at 39 °C, nor do they increase maximal ECAR or lactate production at higher temperatures ^[12]^. Glycolysis is well reported to be key for T cell activation and function. As well as the production of pyruvate and ATP, another important byproduct of glycolysis is the production of nicotinamide adenine dinucleotide (NADH), an essential substrate for CI function. Heterogeneity in NAD(H) biosynthesis underlies cell-to-cell variation in activation strength in both CD4^+^ and CD8^+^ T cells as well as B cells ^[16]^. It is therefore tempting to hypothesize that the increase in glucose uptake in T_H_17 and iTregs is essential to maintain CI activity at higher temperatures. Why these changes would not occur in T_H_1 cells is not clear. An additional factor that may influence the NAD pool in these cells is NAD+ consumption by the DNA-damage enzyme PARP, which in inflammatory macrophages depletes NAD stores ^[17]^. Future studies measuring NAD+/NADH in T_H_1 and T_H_17 cells at either 37 °C or 39 °C to compare the CI substrate availability, CI expression and complex formation would shed light on this question.

Both in this study, and another study in CD8^+^ T cells, febrile temperatures of 39 °C were shown to increase mTOR activation in T cells ^[9,12]^. mTOR is activated by various pathways; however, the exact mechanism of mTOR activation in this context is not clear ^[18]^. Tregs, interestingly, do not rely on glycolysis or mTOR activity as much as their Th cell counterparts ^[19,20]^. This makes it difficult to separate the role of mTOR and CI in T cell function. A previous study, not performed in the context of fever, showed that Rotenone treatment decreased T_H_1 cell proliferation but did not affect IFNγ production ^[21]^. In the current study, Rotenone treatment in T_H_1 cells increased HSP70 and mitochondrial ROS induction; however, IFN*γ* production was not measured in these cells. The balance of mTOR driving T_H_1 responses and CI potentially limiting T_H_1 responses may complicate the interpretation of results with inhibitors.

Another interesting point raised by this study is the concept of environmental T cell selection. Exposure to 39 °C conditions leads to the death of many T_H_1 cells; however, those that survive show increased cytokine production and responsiveness. This parallels another T cell context, which is the generation of memory cells. After effector cell expansion, there is a contraction of activated T cells due to cell death, with the remaining surviving memory cells exhibiting heightened metabolism and responsiveness to subsequent antigen exposure. In fact, precursors of longlived memory cytotoxic T cells were found to exhibit increased resistance to DNA damage ^[22]^. Whether increased temperatures are required for memory responses remains to be explored.

While increased activation of T cells may contribute to a successful antigen-specific response, it also has potential negative consequences, that is, increased damage to the host via enhanced inflammation. Adding to this risk is the increase in somatic mutations induced in these T cells upon DNA damage. Somatic mutations have been associated with antigen spreading and the promulgation of autoimmunity ^[23]^. Indeed, a recent study has identified somatic mutations in CD8^+^ T cells in newly diagnosed rheumatoid arthritis patients ^[24]^. Investigating potential differences in temperature responses and the risk of autoimmunity in different species would be of interest, given the hypothermic febrile response of infected mice compared to the hyperthermic febrile response of humans. This study opens up the possibility of temperature regulation as a pharmacological treatment not just to ease symptoms of inflammation but to prevent potential feed-forward cycles of auto-inflammation.

## Figures and Tables

**Figure 1:**
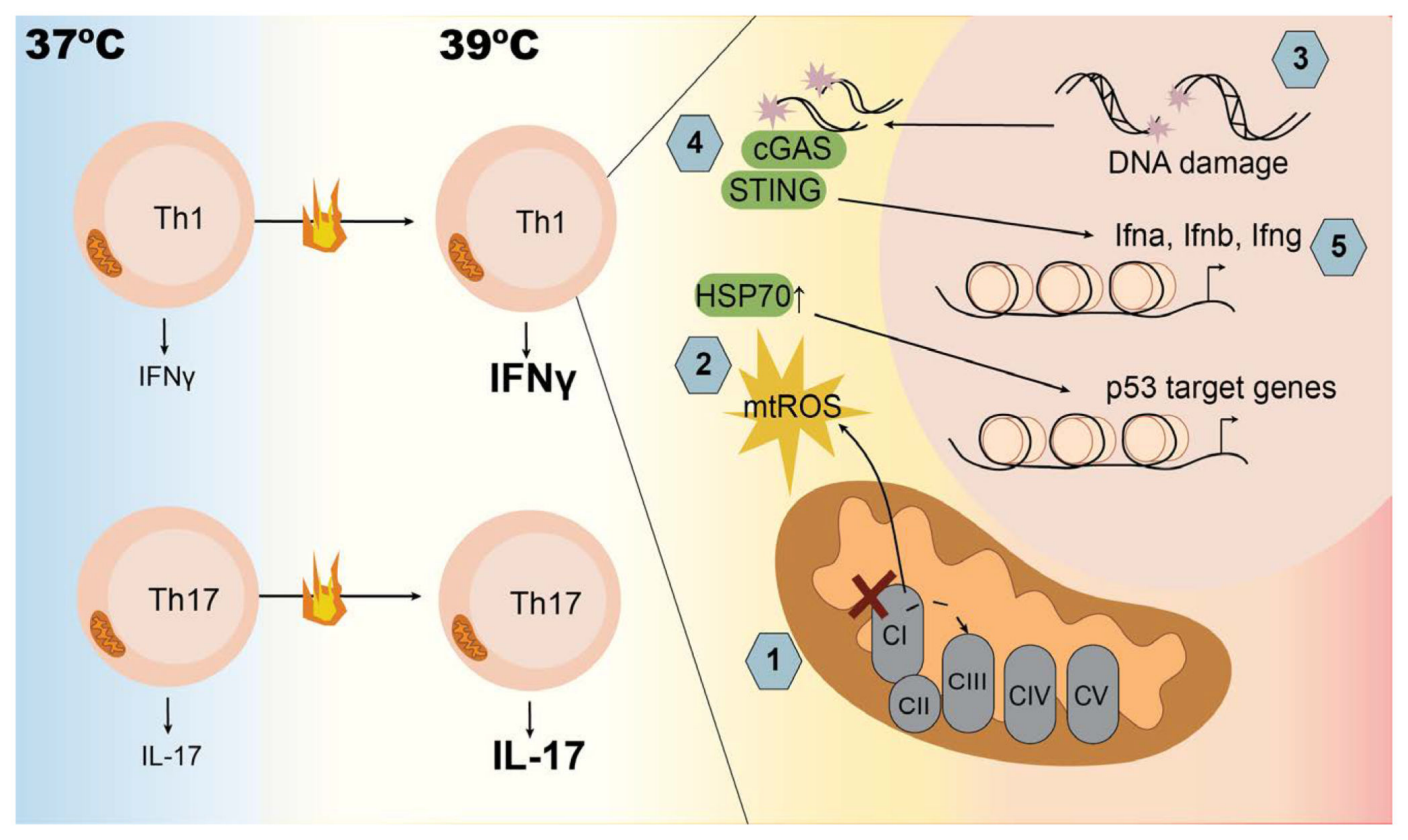
T_H_ cell response to febrile temperatures. T_H_1 cells (upper) cultured at 39 °C display increased production of IFN*γ*. These cells show no increase in glycolysis but instead present mitochondrial complex I dysfunction (1), leading to the generation of mitochondrial ROS production (2), which induces HSP70 expression, DNA damage, and the activation of DNA repair pathways including p53 (3). Damaged DNA activates the cytosolic DNA sensors cGAS and STING to induce Type I and Type II IFNs (4 and 5) as well as promoting further ROS production. This sequence of events explains the increased IFNy production by T_H_1 cells at physiological febrile temperatures. On the other hand, while also producing more of their effector cytokine, IL-17, T_H_17 cells (lower) cultured at 39 °C display no mitochondrial dysfunction and an increase in glycolysis. The mechanism by which T_H_17 cells at 39 °C produce more IL-17 than control cells at 37 °C is not explored in this study. cGAS, cyclic GMP-AMP synthase; STING, stimulator of interferon genes.
